# Appendiceal Mucinous Neoplasm: An Unexpected Diagnosis in Abdominal Pain

**DOI:** 10.7759/cureus.80323

**Published:** 2025-03-10

**Authors:** Inês Pereira Soares, António Sampaio Mesquita, Luís Ramos, Esperança Ussene, Florbela Cunha

**Affiliations:** 1 Paediatrics, Unidade Local de Saúde Estuário do Tejo – Hospital de Vila Franca de Xira, Lisbon, PRT; 2 General Surgery, Unidade Local de Saúde Estuário do Tejo – Hospital de Vila Franca de Xira, Lisbon, PRT; 3 Pathology, Unidade Local de Saúde Estuário do Tejo – Hospital de Vila Franca de Xira, Lisbon, PRT

**Keywords:** abdominal pain, appendicitis, appendix, low-grade appendiceal mucinous neoplasms, mucin

## Abstract

Appendiceal mucinous neoplasms are neoplastic appendicular lesions resulting from the abnormal accumulation of mucin in the appendix. These neoplasms can be asymptomatic or present with vague, nonspecific symptoms. Although generally considered benign, they carry the risk of severe complications such as pseudomyxoma peritonei. We present a 17-year-old boy with fever and suprapubic pain radiating to the right lower quadrant, with no definitive clinical, imaging, or intraoperative macroscopic diagnosis, who was found to have an appendiceal mucinous neoplasm. He underwent complete resection of the appendix with negative margins, without the need for further treatment. As a rare entity in pediatric patients, these neoplasms pose both diagnostic and therapeutic challenges with ongoing debate regarding the best surgical approach, appropriate adjuvant therapy, duration of follow-up, and imaging techniques for their management. This case highlights an unusual way of diagnosing an uncommon neoplasm.

## Introduction

Appendiceal lesions fall into two categories, neoplastic and non-neoplastic, according to the 2019 classification by the World Health Organization [1]. Within the neoplastic category are serrated polyps, hyperplastic polyps, appendiceal mucinous neoplasms (AMNs), which can be classified as low-grade (LAMN) or high-grade [2], and appendiceal goblet cell adenocarcinomas, the recommended terminology for appendiceal neoplasms that were previously classified within the spectrum of carcinoids [3].

AMNs have an incidence of 0.12 per million people per year [1] constituting 0.2-0.3% of cases at appendectomy [4]. Predominantly observed in middle-aged individuals, especially women [5], they are exceptionally rare in the pediatric population [1].

These neoplasms exhibit varied clinical presentations. Approximately 25% of patients are asymptomatic [4], while others may experience only abdominal pain [6]. AMNs are often incidentally detected either during an ultrasound conducted for suspected acute appendicitis or through abdominal imaging performed for other clinical indications [5]. Less frequently observed manifestations include abdominal distention, palpable mass, vomiting, intestinal obstruction, weight loss, and intussusception. Uncommonly, urological manifestations such as ureteral obstruction, hematuria, hydronephrosis, and urinary tract infections may occur [6].

AMNs are initially assessed macroscopically, but accurate diagnosis relies on microscopic examination [7]. The pathogenesis involves abnormal accumulation of intraluminal mucin, leading to abnormal distension of the appendix [4]. A LAMN is confirmed if atypical glandular cells, neoplastic mucinous epithelium, back-to-back crypts, sparse lamina propria, extended villi, and low-grade cytology without infiltrative invasion are identified in pathological examination [6].

Despite being generally considered benign, they pose risks of complications, dependent on tumor size and histological type [6]. Complications can vary from intestinal obstruction/bleeding to spontaneous perforation and peritoneal dissemination, causing pseudomyxoma peritonei [8], a condition associated with a poor outcome [6] and a 10-year survival rate of 45% [7].

With few reported cases of LAMN in children [9], the existing knowledge is largely derived from adult data, and therefore, consensus on appropriate treatment and surveillance for AMNs is lacking. Surgeons vary in their choice of surgical approach and extent of resection for LAMN, and there are no guidelines for surveillance after resection [10].

## Case presentation

A 17-year-old boy, with no significant medical history, presented to the emergency department reporting a one-day history of fever and intense, constant suprapubic pain radiating to the right lower quadrant. There were no concurrent symptoms such as diarrhea, nausea, vomiting, loss of appetite, dysuria, hematuria, urinary frequency/urgency, or urethral discharge. His mother was human immunodeficiency virus (HIV) positive with no other relevant family history. 

On physical examination, he was febrile with an unwell appearance and was unable to walk. Rebound tenderness was observed in the hypogastric and right iliac regions. The remainder review of systems was unremarkable.

Laboratory values were normal including a white blood cell count of 9100/uL (4500-14500/uL), neutrophils of 6810/uL (1500-8000/uL), and lymphocytes of 1460/uL (1500-5000/uL) with a slight elevation of C-reactive protein of 3.51 mg/dL (0.06-1 mg/dL). There were no significant changes in renal and hepatic parameters. The urinalysis yielded normal results. An abdominal ultrasound demonstrated laminar internal fluid, within the hypogastric and right iliac fossa. Inflammatory alterations were observed in the adipose tissue of the right iliac fossa, with no definitive visualization of the cecal appendix. 

**Figure 1:**
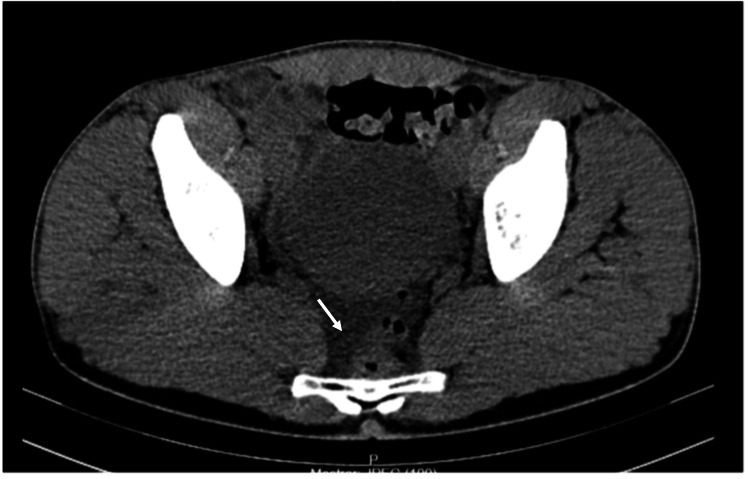
Abdominal computed tomography scan Small amount of intraperitoneal fluid with no visualization of the ileocecal appendix (arrow)

As the visualization of the appendix proved challenging, and following a consultation with the general surgery team, the patient was kept under observation. During this period, a new blood sample was obtained, and an abdominal computed tomography (CT) scan was conducted. The updated laboratory results revealed an increase in C-reactive protein to 11 mg/dL, and the CT scan showed a minor amount of intraperitoneal fluid, yet the identification of the ileocecal appendix remained unattainable (Figure 1).

The adolescent continued to experience pain, more localized to the right iliac fossa, and fever. In the absence of definitive clinical and imaging evidence, a diagnostic laparoscopy was assumed.

During the intraoperative examination, only clear, translucent ascitic fluid was identified. The appendix exhibited no thickening or signs of inflammation. There was an absence of terminal ileitis and thickening or inflammation of ileum loops or Meckel's diverticulum, and no mucin deposits were observed in any organs. However, it was decided to perform a prophylactic appendectomy, and the specimen was submitted for histological assessment.

Following the surgical procedure, empirical intravenous antibiotic therapy with cefotaxime, gentamicin, and metronidazole was initiated. He remained hemodynamically stable, became apyretic after 24 hours, and exhibited progressive clinical and analytical improvement. The ascitic fluid underwent bacteriological examination, which was negative, and showed no presence of acid-fast bacilli. Blood and urine cultures were negative. On the fifth postoperative day, he was discharged and underwent an uneventful recovery. 

**Figure 2:**
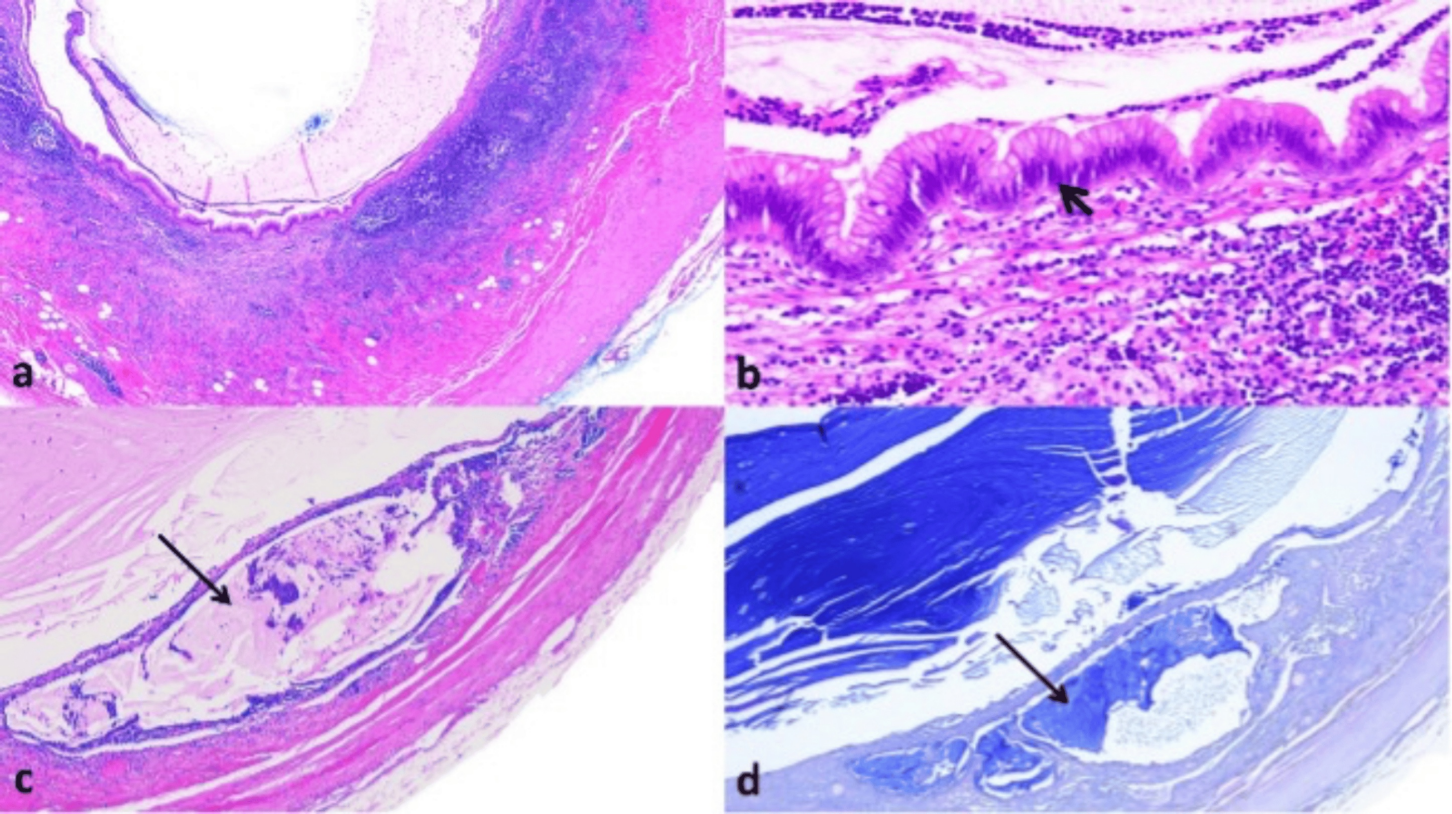
Histopathological sample (a) Appendicular lumen: dilated and filled with mucus. (b) Appendicular lining epithelium: flattened, low-grade atypia (short arrow). (c) Mucus invades the submucosa, without going beyond it (long arrow). (d) Alcian blue staining confirms the presence of mucus in the submucosa (long arrow)

The gross pathological evaluation showed a 5.8-cm-long appendectomy specimen, with intact serosa and a congestive appearance. The lumen was dilated in the proximal portion (1 cm in diameter) and was filled with mucus, which exteriorized through the surgical margin. Microscopic pathologic examination revealed a LAMN with the extension of the mucinous material into the submucosa, pathological staging TisLAMN (Figure 2).

Following consultation with an oncologic team and multidisciplinary discussion, since the diagnosis proved to be a LAMN with negative margins, the appendectomy was considered sufficient treatment, and it was decided to monitor the patient.

He was referred for consultations with oncology and gastroenterology. An abdominal ultrasound was performed for reevaluation, revealing no abnormalities. He is now two years postoperative and doing well with no active complaints. 

## Discussion

AMNs are seldom reported in children [5]. For instance, a study identified only one patient under 18 years of age among 116 cases of AMNs observed over a 30-year period [11]. Additionally, Huang et al. reported on a 16-year-old boy presenting with appendiceal intussusception caused by an AMNs [10].

Since LAMN can often be asymptomatic and present with nonspecific findings when symptomatic, imaging plays a crucial role in their initial detection [6]. This highlights the importance of repeating ultrasound or employing other imaging modalities such as CT scan when there is uncertainty in the diagnosis [1]. However, these imaging modalities have been shown to identify AMNs in only up to 29% of cases [2]. As in this case, the appendix appeared normal, rendering the imaging exams unhelpful.

It is desirable to establish the diagnosis before surgery to mitigate the potential adverse outcome of malignant cell spillage into the abdominal cavity, thus preventing pseudomyxoma peritonei. Nevertheless, a normal imaging result should not postpone surgical intervention [1]. This patient revealed no abnormalities in intraoperative macroscopic examination. The appendix displayed no evidence of dilation or mucocele, which are typically suggestive of the primary diagnosis. The LAMN was solely confirmed through histological analysis; therefore, and considering the rarity of this neoplasm, the diagnosis was unexpected. Given the potential for the lack of macroscopic changes, diagnosing this type of neoplastic lesion becomes even more challenging. Consequently, considering LAMN as a differential diagnosis is crucial when a child or adolescent exhibits symptoms resembling appendicitis [10]. Even when the appendix shows no apparent changes, a prophylactic appendectomy and histological assessment are advisable.

There is ongoing debate surrounding the optimal surgical approach, adjuvant therapy, duration of follow-up, and imaging techniques for managing and monitoring LAMN [2].

Surgical removal of the appendix is essential for treatment, and it can be obtained through open or laparoscopic resection. While open surgery is occasionally advised to reduce the risk of LAMN rupture and subsequent pseudomyxoma peritonei, documented cases of such complications exist for both resections, with no available comparative studies [6]. Literature review suggests that the predominant treatment for LAMN has been open appendectomy, however with a notable demographic trend towards women in their 60s undergoing these resections [1].

For patients classified as low-risk LAMN, McDonald et al. proposed a watch-and-wait strategy, recommending surveillance imaging at six months followed by annual assessments [12]. Gonzalez et al. advised radiographic imaging every six months post-appendectomy for two years and, subsequently, annual CT scans, physical examinations, and monitoring of tumor markers for five to ten years [2]. Tumor markers should include CEA, CA 19-9, and CA-125 [6].

In this case, there was no evidence of malignancy infiltrating the muscularis propria or lymph node metastasis, nor were malignant cells found within the periappendiceal tissue. Consequently, further surgical interventions or adjuvant therapies were deemed unnecessary. 

## Conclusions

This case underscores the significance of maintaining a high level of suspicion for appendiceal tumors in pediatric patients, ensuring that complaints of abdominal pain are not dismissed. This proactive approach helps to diminish long-term complications, which can have a substantial impact on morbidity and mortality.

Further comparative studies in pediatric populations are necessary to ascertain the optimal methods of diagnosis, treatment, and follow-up, aiming to standardize practices and enhance widespread utilization. It is imperative to share all cases of children with LAMN to bolster scientific evidence.
